# Attenuating the ‘attentional white bear’ effect enhances suppressive attention

**DOI:** 10.3758/s13414-022-02560-w

**Published:** 2022-09-22

**Authors:** Alex Muhl-Richardson, Maria Tortosa-Molina, Sergio A. Recio, Maximilian G. Parker, Greg J. Davis

**Affiliations:** 1 Department of Psychology, University of Cambridge, Downing Street, Cambridge, CB2 3EB UK; 2 Department of Communication and Applied Behavioural Science, Royal Military Academy Sandhurst, Camberley, GU15 4PQ UK

**Keywords:** Attention, Suppression, Attentional white bear, Visual search, Eye movements

## Abstract

Trying to ignore an object can bias attention *towards* it – a phenomenon referred to as the ‘attentional white bear’ (AWB) effect. The mechanisms behind this effect remain unclear. On one hand, the AWB may reflect reactive, ‘search and destroy’ distractor suppression, which directs attention toward irrelevant objects in order to suppress further attention to them. However, another possibility is that the AWB results from failed *proactive* distractor suppression – attempting to suppress attention to an irrelevant object from the outset may inadvertently result in an attentional shift towards it. To distinguish these two possibilities, we developed a categorical visual search task that addresses limitations present in prior studies. In five experiments (*N*_*total*_ = 96), participants searched displays of naturalistic stimuli cued only with distractor categories (targets were unknown and unpredictable). We observed an AWB and later attenuated it by presenting a pre-search stimulus, likely disrupting guidance from distractor templates in working memory. We conclude that the AWB resulted from a failure of proactive suppression rather than a search and destroy process.

**Supplementary Information:**

The online version contains supplementary material available at 10.3758/s13414-022-02560-w.

## Introduction

The brain’s ability to suppress attention to objects flexibly and voluntarily is fundamental to its function; however, a seemingly maladaptive feature of this process can cause paradoxical results. When attempting to ignore or suppress attention to an irrelevant object during visual search, attention is often initially biased *toward* it. This counterintuitive effect is informally referred to as the ‘attentional white bear’ (AWB), as the phenomenon parallels Dostoevsky’s (1863/2016) anecdote on the difficulty of suppressing *thoughts* of a white bear (for a review, see Wenzlaff & Wegner, 2000). In the real world, this can affect visual searches where what the target looks like is unknown, but what the target does *not* look like is known. For example, imagine having a nut allergy and looking through a dessert menu for dishes that do not contain nuts, but finding that your attention quickly falls on walnut cake. It is this top-down voluntary attempt to ignore, such as when following instructions, followed by an outcome opposite to that which was intended that sets the AWB apart from other attentional effects.

The AWB highlights a situation in which one might be best served by disregarding additional information about a task. If knowing additional information about irrelevant distractors will result in attention shifting to these distractors, then search might proceed more efficiently or effectively if this remained unknown. Furthermore, it is important to note that the AWB is distinct from: (1) effects related to the suppression of bottom-up attentional capture by salient objects (Gaspelin et al., 2017; Sawaki & Luck, 2010; Theeuwes & Godijn, 2002), (2) cases of spatial attention in which stimuli appearing *at particular locations* are known to be irrelevant (Noonan et al., 2016), and (3) increases in alertness associated with expecting or preparing for distractors (Makovski, 2019).

The literature on the suppression of attention to distractors has highlighted two different suppression mechanisms that provide distinct contexts for the AWB. One of these mechanisms is reactive suppression, which involves initially *searching for* distractors, locating them, and then suppressing attention to them. While this requires that distractors are first attended to before they can be ignored, importantly, it need not involve a deliberate decision to attend to the distractor. It may be the case that the AWB reflects nothing more than a reactive suppression process.

The possibility that the AWB arises from correctly functioning reactive suppression was first identified by Tsal and Makovski (2006), who investigated the AWB using a flanker task with infrequent dot and line probe trials. They found that participants made early shifts of attention to expected distractor locations and that these locations were subject to later inhibition. Subsequent studies by these authors showed that this effect was robust to varying task demands, including high perceptual and memory load (Lahav et al., 2012), and that it could not be explained by a simple location priming account (Lahav & Tsal, 2013).

In related work, Moher and Egeth (2012) investigated reactive suppression (which they described as a ‘search and destroy’ process) using a simple visual search task in which in participants were cued to the colour of one distractor and then searched displays of four items for a target that was either a ‘B’ or an ‘F’. They found that a distractor colour cue was associated with slower and less accurate search relative to an uninformative cue, because a distractor cue reliably caused participants to attend to the matching distractor before the target. These results extend previous work by Tsal, Makovski and Lahav (Lahav et al., 2012; Lahav & Tsal, 2013; Tsal & Makovski, 2006), demonstrating an AWB during visual search and illustrating how it can hinder the search process. Together, these studies suggest the possibility that the AWB is simply a necessary component of reactive suppression.

An alternative explanation for the AWB involves a different mechanism – proactive suppression – in which attention toward distractor objects is suppressed from the time of, or even before, their onset (Gaspelin & Luck, 2019; Geng, 2014). Relative to reactive suppression, proactive suppression is more likely to minimise interruption and provide optimal behavioural outcomes; however, it is also more cognitively demanding and may not always be possible due to both individual and task factors (Geng, 2014; Geng et al., 2019). As attending to distractors is not required for proactive suppression, it may be the case that the AWB reflects a failed attempt to proactively suppress. The overall goal of the present study was to differentiate between these two accounts of the AWB by seeking evidence for the AWB as either a necessary component of reactive, search and destroy, distractor suppression or an unintended consequence of failed proactive distractor suppression.

### The AWB versus bottom-up attentional capture

A large portion of the literature on proactive suppression concerns the suppression of bottom-up attentional capture by salient stimuli. As mentioned earlier, attentional capture by salient stimuli is a distinct effect from the AWB, but the discussion of proactive suppression within this literature provides useful context for the present work. In several behavioural and eye movement experiments using visual search tasks, Gaspelin et al. (2015, 2017) demonstrated that salient colour singleton distractors could be proactively suppressed when participants were given a specific target shape to search for rather than simply trying to find a shape singleton target. In a related study, Gaspelin and Luck (2018b) also found that proactive suppression of colour singleton distractors improved when they were a predictable colour and after participants had gained some experience of their appearance. These results build on similar earlier work by Vatterott and Vecera (2012), and are consistent with participants developing templates based on learned distractor features (in these examples, colour) that facilitate proactive suppression.

Further evidence in support of the role of distractor templates (or ‘templates for rejection’) in proactive suppression comes from a study by Won and Geng (2018). They used a visual search task with two stages – a training stage, where participants were exposed to a set of coloured distractors, and a later test stage, which introduced new distractor colours at specific equally spaced points in colour space. Response times for test displays with new distractor colours were consistent with the use of broad distractor templates that could be generalised to suppress similar untrained colours. A later study by the same authors examined the extent to which mere exposure to distractor colours in a task-free context could facilitate the suppression of matching distractors in a separate search task (Won & Geng, 2020). Response time results showed that passive habituation over time improved distractor suppression, possibly by forming the basis of an early attentional filter or leaving participants better prepared to quickly develop and employ distractor templates.

### Target and distractor templates

In the context of proactive suppression, we have discussed evidence that shows distractor templates can be constructed over time based on learned features (Gaspelin & Luck, 2018b; Won & Geng, 2020), that such templates can facilitate the proactive suppression of attention to distractors that broadly match these features (Won & Geng, 2018), and that this is an effortful process (Geng, 2014; Geng et al., 2019). In contrast to proactive suppression, for reactive suppression, distractor templates function as target templates, positively guiding attention to distractors so that they can be rejected (Moher & Egeth, 2012).

Arita et al. (2012) conducted a study that directly compared the use of distractor and target templates using a visual search task where participants were cued with the target colour, one of two distractor colours, or a neutral colour that was never present in search displays. Target and distractor colour cues provided participants with a feature that could form the basis of either a target or distractor template (the neutral cue provided a baseline) and colours were randomly selected from seven possible colours to remove the possibility that the target colour could be inferred from the distractor colour (and vice versa). They found that distractor cues were associated with faster response times than neutral cues and that target cues were associated with faster response times than distractor cues. Unfortunately, these results are limited in the extent to which they can draw out functional differences between distractor and target templates. One explanation is that target and distractor templates were used to set weights within some form of attentional map, but that the difference in the weighting between targets and distractors was not as large as might be expected. Another explanation of the observed disparity between target and distractor cues is that distractor cues supported reactive suppression, with distractors being selected then suppressed.

A subsequent study highlighted that this result might be an artefact of the hemifield split in the stimulus displays, a design that could allow participants to quickly avoid the irrelevant half of the display by translating a target or distractor cue into a simple spatial template (Beck & Hollingworth, 2015). This possibility was addressed by Carlisle and Nitka (2019), who replicated the original task by Arita et al. (2012), but examined the N2pc event-related potential (ERP) component as a measure of which hemifield was attended. Their results showed that attention was directed to the target hemifield following both target cues and distractor cues, but that this shift happened around 150 ms later following distractor cues. Attention was never directed to the distractor hemifield, ruling out the possibility of reactive suppression. In another experiment, Carlisle and Nitka (2019) used a modified design that included interleaved trials with spatially mixed displays (in addition to the original hemifield displays). They found the same results for both types of trial, suggesting that participants adopted a single strategy that was not dependent on ignoring one hemifield.

One further problem highlighted by Becker et al. (2016), related to Beck and Hollingworth’s (2015) suggestion that the colour cues could be translated into spatial templates, is the possibility that target features can be rapidly inferred or predicted even if participants are only shown a distractor cue. All of the visual search experiments discussed in the preceding paragraphs are typical of the broader literature in that they use simple shapes and letters as stimuli. The key advantage of simple stimuli is that they are easily controlled and manipulated experimentally; however, they also limit the extent to which distractor suppression can be isolated from guidance *towards* targets during search. With simple stimuli, even if participants are only shown a distractor cue, it is likely that target features will be more easily inferred or predicted, consistent with evidence from ‘odd-one-out’ search for unknown targets (Found & Müller, 1996; Müller et al., 1995). If participants who are given distractor cues are able to effectively infer or predict their own target templates, either rapidly on a trial-by-trial basis or more slowly over many trials, then their performance will reflect guidance towards targets, potentially to a greater extent than distractor suppression.

### The present study

In the present study, we addressed the problem of predicting or inferring target features using a categorical visual search task with photographic everyday object stimuli (Daffron & Davis, 2015, 2016). Categorical search tasks have been widely used within the visual search literature; for example, they are particularly well suited to investigating effects related to target specificity, typicality and similarity (for a recent review, see Zelinsky et al., 2020). In one such study, Schmidt and Zelinsky (2009) used a categorical search task with photographic stimuli to examine the guidance afforded by different levels of target cue specificity. They found that search guidance was proportional to the informational value of the cues given. Pictorial cues were associated with the greatest proportion of initial eye movements towards targets, with word cues with colour information (e.g., ‘brown boots’, ‘brown footwear’), precise word cues (e.g., ‘boots’) and abstract word cues (e.g., ‘footwear) all providing progressively reduced guidance in that order. These findings suggest that even abstract word cues can provide effective search guidance, but that there is more scope for errors in forming target templates on the basis of broad or abstract category cues.

More recently, Robbins and Hout (2020) investigated how the categorical typicality of targets influences guidance and target verification during search, in the context of both precise and abstract word cues and different levels of target-distractor similarity. They found that more category typical targets were consistently associated with improved attentional guidance when searchers were given a superordinate level abstract cue (e.g., ‘clothing’) and distractors belonged to other superordinate categories. They also found that greater typicality only improved target verification when searchers were given a precise cue (e.g., ‘pants’) and distractors were from the same superordinate category as the target (e.g., other items of clothing). These findings clearly disambiguate the effects of target category typicality and target/non-target similarity on categorical search for precise and abstract cue types, while also distinguishing between the impact of these factors on guidance and target verification.

In addition to being well suited to addressing questions asked by studies like these, a categorical search task offers another critical advantage for the present study. We chose to use a categorical search task with photographic stimuli, because it allowed us to provide participants with broad distractor cues (e.g., ‘keys’ or ‘clocks’), but to select targets from a diverse, unspecified and unpredictable set of categories. This ensured that it was not possible to infer or predict the categories or features of targets and that participants relied only on distractor cues for guidance. While atypical of search tasks in the AWB literature, the necessary imbalance in the relative numbers of target and distractor categories was, for this reason, a crucial feature of the present task. We also note that the task did not *require or encourage* attention to distractors, as participants could determine whether an item was a target or a distractor by whether or not it matched the cued distractor category.

Using this task, Experiment 1 tested for the presence of an AWB under explicit instructions to proactively suppress specific cued distractor categories. We examined participants’ eye movements and analysed the first saccade made when searching each display, specifically its latency and landing position. A greater proportion of first saccades towards distractors than toward targets would provide evidence of an AWB effect. While eye tracking cannot monitor covert attention, our task design and stimulus complexity gave us confidence that eye movements (i.e., overt attention) and covert attention would remain highly correlated here.

Our first aim was simple: to detect an AWB in initial saccades. We predicted that an AWB would be observed when participants were instructed to proactively suppress attention to specific distractor categories. To provide a baseline, we also sought to compare the patterns of initial saccades under these conditions – where participants were instructed to *ignore* specified distractor categories, to conventional search – where participants were instructed to *search for* the same categories of objects. Our task design ensured that we could do so using exactly the same displays. In subsequent experiments, we sought evidence that would support an account of the AWB as either a necessary step in search and destroy suppression or as an unintended failure of proactive suppression. We reasoned that if we could flexibly disrupt or eliminate the AWB, while not impairing the ability to find targets, then this would support an account of the AWB as a failure of proactive suppression and not a necessary component of a search and destroy process.

## Experiment 1

In Experiment 1, we sought to establish the presence of an AWB. One group of participants were given conventional instructions to search *for* specific target categories (‘find’ instructions) and a second group were explicitly instructed to proactively *ignore* specific distractor categories (‘ignore’ instructions). We reasoned that if proactive suppression functioned effectively, the instruction to ignore would result in more first saccades being toward targets than toward distractors. On the other hand, a failure of proactive suppression would be evident in an AWB, with more first saccades towards distractors, particularly at low latencies for rapid reactive suppression. We predicted that we would observe an AWB and that the patterns of eye movements between find and ignore instructions would therefore mirror one another.

### Method

#### Participants

Thirty-two participants took part in Experiment 1 (23 females; nine males; *M*_age_ = 24.16 years; *SD* = 4.37; age range: 18–31 years). Data from one additional participant were excluded due to a failed eye-movement recording. Here, we were primarily interested in large effects (d ≈ 0.8, η2_G_ ≈ 0.2) for which our primary comparisons (two-tailed one-sample *t*-tests, ANOVA) require 16 participants to achieve 80% power (G*Power 3.0; Faul et al., 2007); for our least powerful comparisons (between-participants ANOVA main effects) this analysis suggested 79% power. Our effects of interest proved to be of this size or larger and were robust across experiments, increasing our confidence that these sample sizes were appropriate. Participants were recruited via the Department of Psychology Research Sign-up System at the University of Cambridge and were each compensated with £5 for their time. All participants self-reported normal (or corrected-to-normal) visual acuity and gave informed consent. The experiment was approved by the Cambridge Psychology Research Ethics Committee and conducted in accordance with the Declaration of Helsinki (2013).

#### Apparatus and stimuli

Stimuli were displayed on a 22-in. Dell LCD monitor (1,920 px × 1,080 px resolution, 60-Hz refresh rate) using E-Prime 2.0 in conjunction with an SR Research Eyelink 1000 eye tracker. Calibration was accepted only when none of the five points had an error of more than 0.5° of visual angle. Participants viewed the display binocularly from a distance of 70 cm in a chin rest and only the right eye was tracked.

**Fig. 1:**
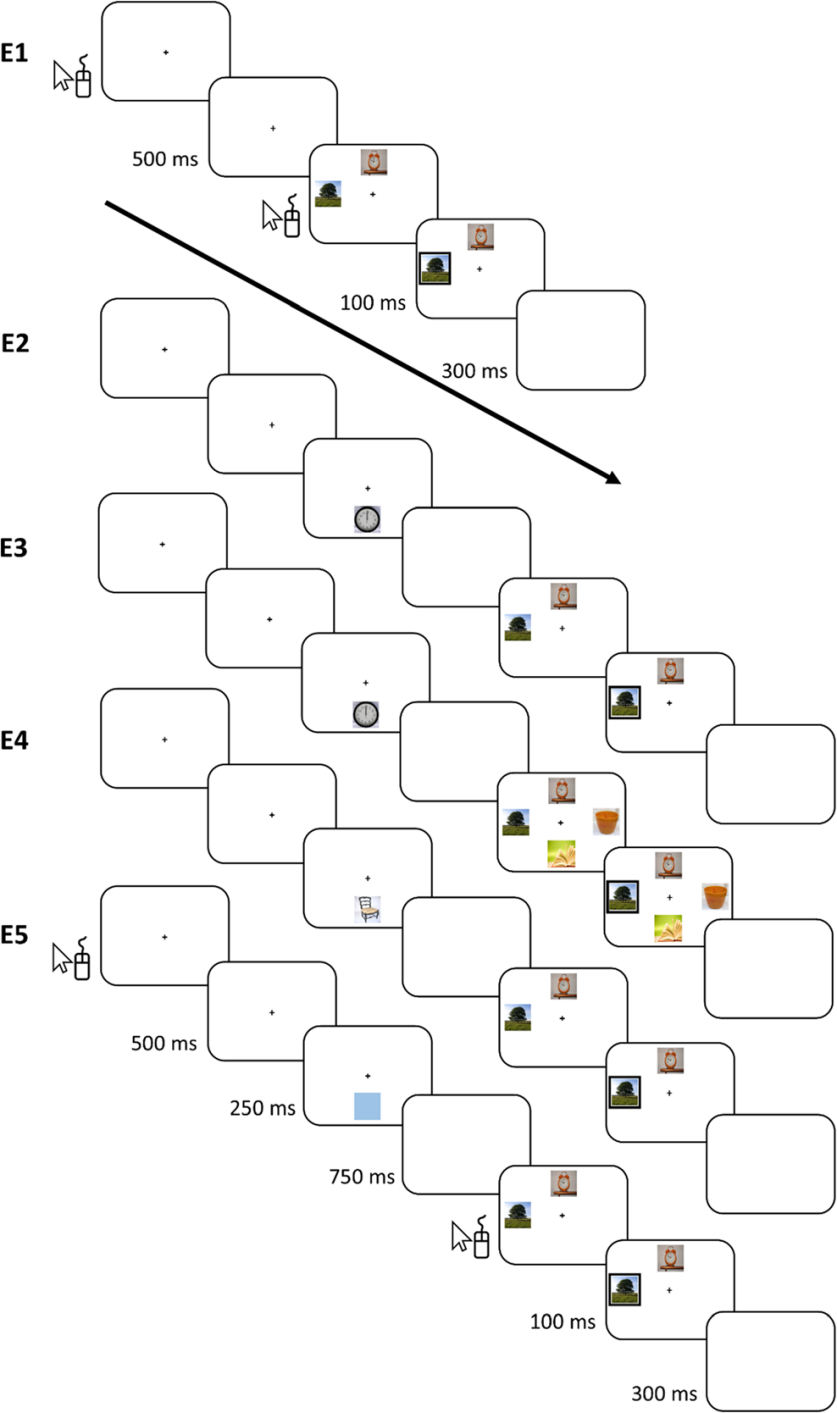
Trial sequences for all experiments (images not to scale), Experiments 2–5 had identical timings and responses (see Table 1 for a summary of experimental methods)

Stimuli were square photographic images of real-world objects, each 6.09° of visual angle. There were four possible locations in which stimuli could appear and these were centered 7.79° of visual angle above, below, right and left of the display centre (see Fig. 1). For ignore instructions, 72 images of clocks and 72 images of keys were used as distractors and 144 images of other categories of object were used as targets, such that target category was not predictable or inferable from distractors. For find instructions, exactly the same displays were used, but distractors from the ignore condition were now designated as targets and targets as distractors. Trial order was randomized within blocks and the positions of target and distractors were counterbalanced.

#### Procedure

Participants were assigned to one of two groups which received instructions to either proactively ignore a specified distractor category and respond directly to the target (ignore group) or to find a specified target category and respond to it (find group). No additional information was given to participants in the ignore group about targets. The experiment consisted of 144 trials in four equal-length and order counterbalanced blocks, with a self-paced break at the midpoint. For the ignore group, two blocks included a single distractor category (either clocks or keys) and two blocks included two distractor categories (clocks and keys, one of either category on each trial). For the find group, two blocks included a single target category (either clocks or keys) and two blocks included two target categories (clocks and keys, one of either category on each trial)

Each block began with on-screen instructions and exact instructions are reported in the Online Supplementary Material (OSM). These instructions stated that each trial would show one target and one distractor, each in one of four positions – to the right of, left of, above or below the central fixation cross. Participants were informed of the specified category and whether they should ignore or find stimuli in that category. They were also reminded that, while the stimuli would be different between trials, one image would always belong to that category.

In each trial, participants were required to use the mouse to click on a central fixation cross to proceed, the cross then remained present for 500 ms, and then a trial display of one target and one distractor was shown until the target was clicked. Following a target click, a black border appeared around the target for 100 ms to indicate a registered response, then a blank screen was displayed for 300 ms before the next trial. The trial sequence is shown in Fig. 1 and a procedural summary for all experiments is shown in Table 1.

**Table 1:** Summary of experimental methods

Experiment	Instruction	Pre-search stimulus	Trial display	NT categories per block
1	Ignore/find*	No	1 T & 1 D	1 or 2 (T categories for find)†
2	Ignore	Yes (D congruent)	1 T & 1 D	1 or 2†
3	Ignore	Yes (D congruent)	1 T & 1/3 Ds†	1
4	Ignore	Yes (D congruent/incongruent, interleaved)†	1 T & 1 D	2
5	Ignore	Yes (colour)	1 T & 1 D	1

Target (T), distractor (D); * = manipulated between-participants; † = manipulated within-participants

### Results

#### Analytic approach

**Fig. 2:**
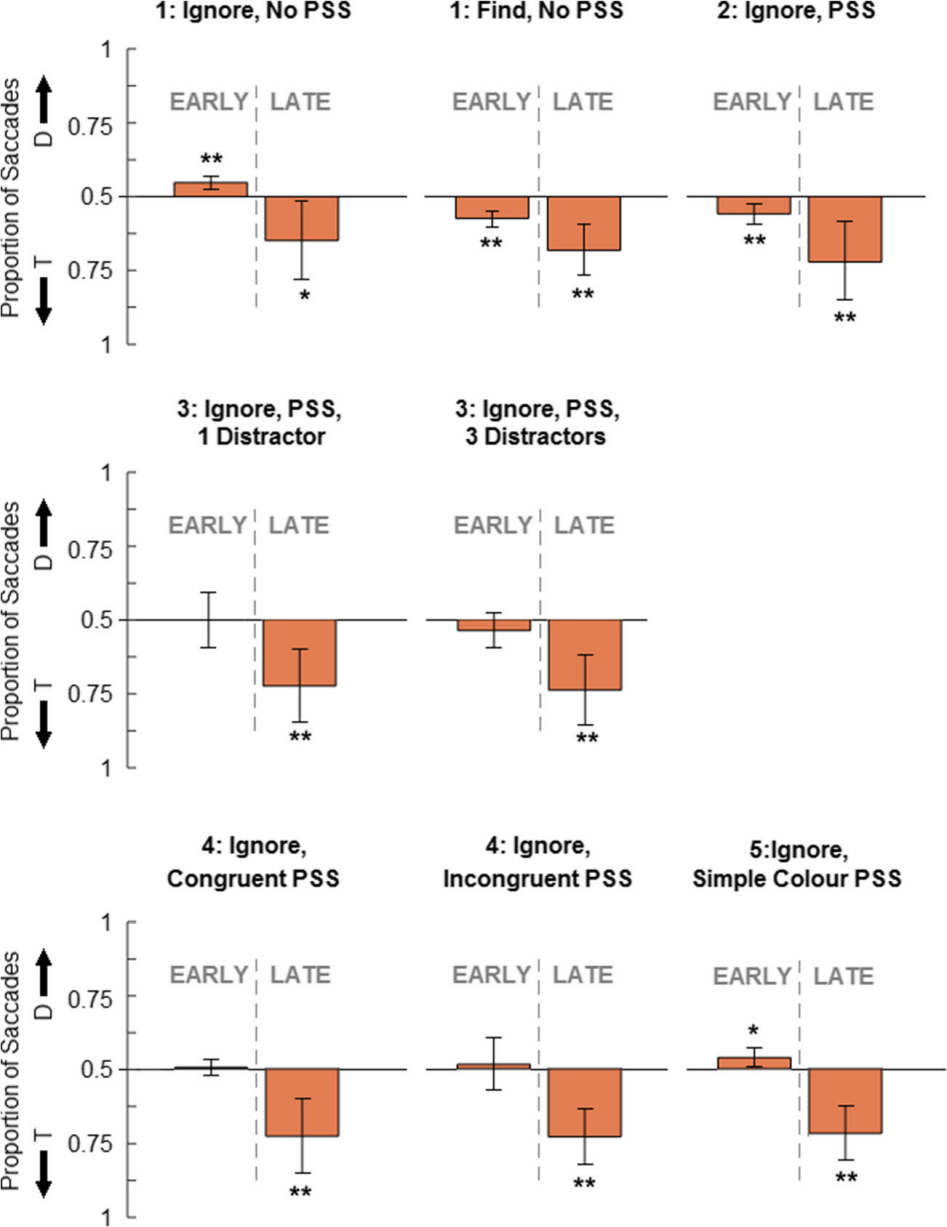
Bar charts (95% confidence interval error bars; asterisks indicate one-sample *t*-tests, *μ* = 0.5, * *p* < .05, ** *p* < .01) of proportions of early (< 250 ms) and late (> 250 ms) first saccades to distractors (D) and targets (T), averaged across participants. In the ‘ignore’ instructions condition of Experiment 1 (top row, left) first saccades were fast and there was an early bias toward distractors (AWB effect), under typical search instructions (‘find’, top row, middle), a target bias was observed in both early and late saccades. In Experiment 2 under ignore instructions (top row, right), the introduction of the pre-search stimulus (PSS) extinguished this early bias. Experiment 3 produced similar results to Experiment 2 in displays with one (middle row, left) and three (middle row, right) distractors. No effect of PSS category congruency was observed in Experiment 4 (bottom row, left and middle). In Experiment 5, with a simple colour PSS (bottom row, right), an early target bias was observed as in Experiment 1 under ignore instructions

We examined the first saccades made to targets and distractors after search display onsets. In all experiments presented here, our analysis only included saccades initiated at least 70 ms after the onset of the search array (to exclude anticipatory saccades). We used four equally sized trapezoid regions to code saccade landing positions (all shared one side of a 104-px square around the central fixation cross and had a longest side of 1,080 px). We also examined differences in the timing of first saccades using a simple cut-off at an onset latency of 250 ms, an estimated upper-bound to the range of normal saccade latencies expected in simple and real-world visual search tasks (Cronin et al., 2020; Darrien et al., 2001; Findlay, 1997) and consistent with the distribution of saccade latencies observed in Experiment 1. Our principal analyses were a set of planned one-sample *t*-tests to compare the proportion of first saccades made to distractors (out of the total first saccades to targets and distractors) for late compared to early saccade latencies at the level of each instruction (*μ* = 0.5, which indicates no target/distractor bias; also see Fig. 2 and Table 2 for descriptive statistics). These analyses were collapsed across categories, but see OSM for additional analysis.

**Table 2:** Descriptive statistics

Experiment	Condition	Mean RT (ms)	Mean first saccade latency (ms)	Mean fixation durations (ms)			Mean proportions of first saccades to distractors	
				Overall	Distractor	Target	Early	Late
1	Ignore	792 (140)	167 (44)	266.23 (45.53)	189.55 (55.42)	306.19 (56.26)	0.55 (0.04)	0.34 (0.26)
	Find	715 (128)	172 (35)	263.71 (20.29)	177.11 (27.47)	310.63 (32.86)	0.42 (0.05)	0.32 (0.16)
2	Ignore (PSS)	805 (79)	265 (104)	282.55 (39.81)	186.62 (28.66)	328.64 (54.19)	0.44 (0.06)	0.29 (0.23)
3	Set size 2 (PSS)	835 (102)	307 (118)	301.12 (54.10)	191.39 (44.85)	349.12 (68.12)	0.50 (0.17)	0.27 (0.23)
	Set size 4 (PSS)	1,067 (110)	339 (153)	257.38 (42.42)	174.20 (19.24)	329.01 (59.09)	0.46 (0.11)*	0.26 (0.22)*
4	Congruent PSS	846 (126)	252 (106)	296.05 (50.44)	189.15 (26.15)	350.14 (61.03)	0.51 (0.05)	0.27 (0.24)
	Incongruent PSS	853 (116)	252 (99)	290.93 (41.24)	203.24 (29.62)	338.47 (50.02)	0.52 (0.17)	0.27 (0.18)
5	Colour PSS	851 (126)	240 (85)	287.91 (56.91)	185.29 (29.45)	346.46 (67.86)	0.55 (0.07)	0.36 (0.14)

*Note:* Mean proportion of first saccades to targets equals one minus the values shown in the rightmost column, early first saccades were those with latency < 250 ms, late first saccades were those with latency > 250 ms, parentheses show standard deviations; * = adjusted to maintain target-distractor equivalence (target frequencies multiplied by three); PSS = pre-search stimulus

#### Ignore instructions

Under ignore instructions, there was a greater proportion of early (< 250 ms) first saccades made towards distractors than targets, *t*(15) = 4.43, *p* < .001, *d* = 1.11, and a greater proportion of late (> 250 ms) first saccades made towards targets than distractors, *t*(15) = 2.35, *p* = .033, *d* = 0.59, (see Fig. 2, top row, left). This indicates a strong AWB effect in early first saccades for participants who were given ignore instructions.

To confirm that the AWB observed here reflected the instruction to ignore a specific distractor category and not the intrinsic properties of the stimuli themselves (e.g., differences in luminance contrast or colour), we contrasted these results with a baseline experiment. This used the same stimuli but with no instruction to ignore and yielded no evidence for a distractor or target bias in the time window (< 250 ms) corresponding to the AWB we detected (see OSM).

#### Find instructions

We conducted matching one-sample *t*-tests for find instructions and found greater proportions of both early (< 250 ms), *t*(15) = 5.92, *p* < .001, *d* = 1.48, and late (> 250 ms), *t*(15) = 4.51, *p* < .001, *d* = 1.13, first saccades made towards targets than distractors (see Fig. 2, top row, middle). For participants who received find instructions, both early and late first saccades were more likely to be made towards targets. The observed effect for early first saccades towards targets here mirrored that observed for early first saccades made towards distractors for ignore instructions.

#### Effects across instructions

**Fig. 3:**
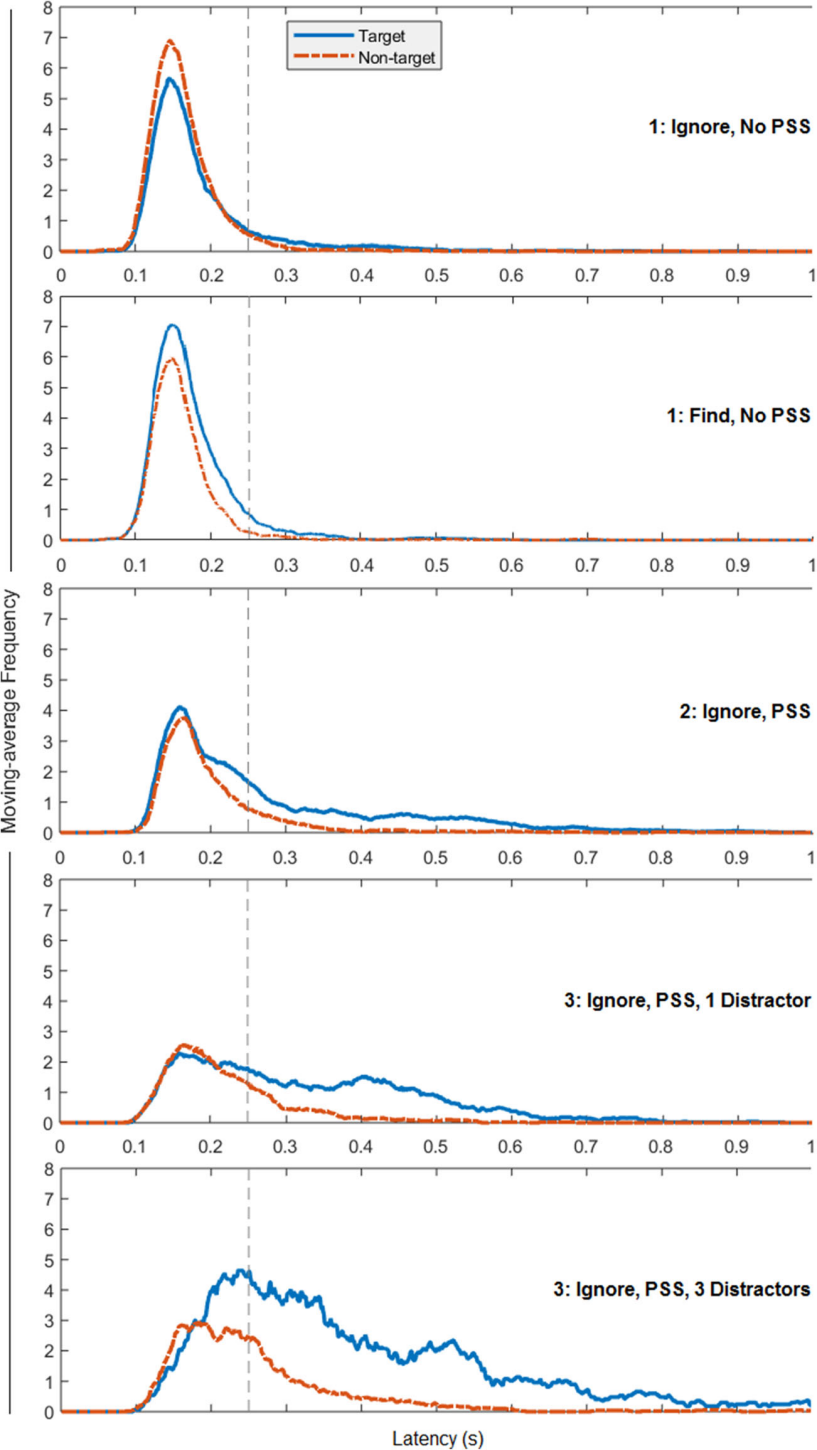
Moving-average frequency of first saccades made to targets and distractors in Experiments 1–3, calculated over onset latencies for all first saccades. The top two plots show data from the ‘ignore’ and ‘find’ instruction conditions from Experiment 1 where no pre-search stimulus (PSS) was present. The middle plot shows data from Experiment 2 where ignore instructions and a PSS were used. The bottom two plots show data from Experiment 3, which replicated the conditions of Experiment 2 but with one and three distractor displays. The vertical dashed line indicates the 250-ms onset latency cutoff used to categorise early and late first saccades

Figure 3 (top and second from top) shows the number of initial saccades made towards distractors versus targets as moving averages over time and, consistent with our primary analyses, visual inspection suggests the presence of an AWB (a bias for attending to distractors) for ignore instructions. To examine differences between instruction conditions and to supplement our earlier analyses, we also examined the proportion of first saccades to distractors (out of the total first saccades to targets and distractors) using a two-way mixed ANOVA with first saccade latency (within-participants: early (< 250 ms), late (> 250 ms)) and instruction (between-participants: ignore, find) as factors.

Our primary analyses were focussed on determining whether more first saccades were made towards targets or distractors by testing each condition for differences from an equal proportion of target and distractor first saccades. We now address a different aspect of these data by examining differences between each level of the two factors here rather than deviation from an equal proportion of target and distractor first saccades. While our initial analyses indicated that the only combination of conditions where more first saccades were made towards distractors than targets was at early latencies under ignore instructions, we did not necessarily expect an interaction between the effects of instruction and first saccade latency in this ANOVA. Our earlier analyses would be consistent with any result where only one level of one factor showed a greater proportion of distractor first saccades. In this case, the ANOVA revealed a main effect of saccade latency, *F*(1,30) = 14.43 *p* < .001, η2_G_ = .20, indicating a greater bias toward targets in late saccades. There was no effect of instruction, *F*(1,30) = 3.68, *p* = .065, and no interaction, *F*(1,30) = 1.46, *p* = .236.

#### Further exploratory analysis

We conducted further exploratory analyses to better contextualise the effects described above (see Table 2 for descriptive statistics, *p*-values Bonferroni-corrected for the three exploratory comparisons on these data). We examined the effects of instruction on overall response times and first saccade latencies. There was no effect of instruction on response time, *t*(30) = 1.63, *p* = .339, or on first saccade latency, *t*(30) = 0.44, *p* > .999. We also examined the effect of instruction on fixation durations, including overall, distractor and target fixation durations. There was no effect of instruction on overall, *t*(30) = 0.20, *p* > .999, distractor, *t*(30) = 0.80, *p* > .999, or target, *t*(30) = 0.27, *p* > .999, fixation durations.

### Discussion

Participants who received ignore instructions were unable to voluntarily engage in effective proactive distractor suppression despite being explicitly instructed to do so, demonstrating a strong AWB effect in early first saccades. In late first saccades, a marginal bias towards targets was observed, which suggests that overcoming the early distractor bias was sometimes possible. Participants who received find instructions showed a consistent bias towards targets in both early and late first saccades, demonstrating that they were able to voluntarily attend to the specified target categories independently of first saccade latency. The low overall average latency of first saccades strongly suggests that, as predicted, overt and covert attention remain highly correlated in the present case.

Having observed a broadly consistent pattern of initial saccades (as well as response times and fixation durations) across instruction conditions in Experiment 1, we wished to explore whether the observed AWB was a necessary component of suppression in the current task by testing conditions under which this effect might be eliminated. Previous work has demonstrated that repeated exposure to distractors over many blocks of trials can reduce the AWB (Cunningham & Egeth, 2016). We wished to compare search processes without the influence of habituation or extended practice and sought to eliminate the observed AWB effect rapidly and without changing the task. We aimed to test whether an additional presentation of a stimulus from the distractor category prior to the onset of the search array (a pre-search stimulus) might impact the AWB. If it is the case that holding a distractor template in working memory (WM) biases early attention towards matching objects (Han & Kim, 2009; Sawaki & Luck, 2011; Woodman & Luck, 2007) and contributes to the AWB, then a brief presentation of an additional stimulus immediately prior to the search display might disrupt this. In Experiment 2, we therefore sought to establish whether a pre-search stimulus could disrupt the early tendency to attend to distractors and eliminate the AWB effect observed in Experiment 1.

## Experiment 2

Experiment 2 replicated the conditions under ignore instructions in Experiment 1 with the addition of a pre-search stimulus for each trial that briefly appeared prior to the search display. The pre-search stimulus was always an item from the same category as the distractor in the subsequent search display. We predicted that the early tendency to attend to distractors that we observed in Experiment 1 would be disrupted by the pre-search stimulus and that we would not observe the AWB in terms of early first saccades to distractors.

### Method

#### Participants

Sixteen participants took part in Experiment 2 (11 females; five males; *M*_age_ = 23.19 years; *SD* = 5.19; age range: 19–41 years). Recruitment, compensation, visual acuity, consent and ethical approval were subject to the same criteria as in Experiment 1.

#### Apparatus and stimuli

The apparatus and stimuli used in Experiment 2 were identical to those used in Experiment 1 with the following exceptions. The trial displays in Experiment 2 were preceded by a single pre-search stimulus, selected from a set of 72 images of clocks and 72 images of keys, appearing in one of the same four locations (and the same size) as the search stimuli. Pre-search stimuli were always drawn from the same category as the distractor in each trial, but were never identical to the distractor, and each was only presented once. In Experiment 2, the location of the pre-search stimulus was controlled and never appeared in the same location as the target in the subsequent search display.

#### Procedure

The procedure was the same as for Experiment 1, with the exception that in each trial, a pre-search stimulus was displayed for 250 ms, followed by a blank screen for 750 ms, prior to the search display onset (see Fig. 1). There was no manipulation of instructions; all participants were explicitly instructed that the pre-search stimulus was not task-relevant and to ignore it.

### Results

#### First saccade latency

Patterns of first saccades from Experiment 2, plotted in Figs. 2 and 3, suggested that the previously observed AWB was not present. The same analytic approach as in Experiment 1 (one-sample *t*-tests, *μ* = 0.5 indicating no target/distractor bias) revealed a greater proportion of first saccades made towards targets for both early (< 250 ms), *t*(15) = 4.16, *p* < .001, *d* = 1.04, and late first saccades (> 250 ms), *t*(15) = 3.60, *p* = .003, *d* = 0.90. The previously observed bias toward the distractor in early first saccades was not present and instead there was a target bias, consistent with proactive rather than reactive suppression.

#### Pre-search stimulus

To compare the effect of the presence of the pre-search stimulus on early (< 250 ms) first saccades, we conducted an additional comparison between the ignore instruction conditions in Experiment 1 and Experiment 2. The proportion of early first saccades towards the distractor was significantly lower in Experiment 2 (with pre-search stimulus) than in Experiment 1 (no pre-search stimulus), *t*(30) = 5.97, *p* < .001, *d* = 2.11.

#### Further exploratory analysis

To better understand the effect of the pre-search stimulus, we compared overall response times and first saccade latencies between the ignore instruction conditions in Experiment 1 and Experiment 2 (see Table 2, *p*-values Bonferroni-corrected for the three exploratory comparisons on these data). There was no effect of the pre-search stimulus on response time, *t*(30) = 0.33, *p* > .999, but the presence of the pre-search stimulus in Experiment 2 was associated with longer first saccade latencies, *t*(30) = 3.52, *p* = .003, *d* = 1.23. We also compared fixation durations between the ignore instruction conditions in Experiment 1 and Experiment 2, including overall, distractor and target fixation durations (see Table 2). There were no differences in overall, *t*(30) = 1.08, *p* = .867, distractor, *t*(30) = 0.19, *p* > .999, or target, *t*(30) = 1.15, *p* = .777, fixation durations.

### Discussion

These results showed that not only was the AWB effect from Experiment 1 eliminated with the inclusion of a pre-search stimulus, but also that participants instructed to ignore distractors were better able to engage in proactive suppression. Further, a greater proportion of early (and late) first saccades were made towards targets than distractors, suggesting that eye movements and covert attention remained highly correlated in the present task. The results of Experiment 1 were consistent with attentional guidance *toward distractors* being elicited by the instruction to ignore them; in marked contrast, the findings of Experiment 2 were consistent with the pre-search stimulus disrupting this guidance, as evidenced by longer first saccade latencies. Response times and fixation durations were consistent between the ignore condition of Experiment 1 and Experiment 2, suggesting that the pre-search stimulus did not impact later perceptual recognition of distractors or targets. It is not clear from the results of Experiment 2 whether these effects required the pre-search stimulus to be drawn from the same category as the distractor or to offer some information regarding its likely position. Our subsequent experiments extend our investigation to displays comprising four stimuli and pre-search stimuli from other categories to reveal that neither of these conditions was required.

## Experiment 3

Experiment 3 included search displays of a larger set size (four stimuli per display) and allowed the pre-search stimulus to appear in any of the four stimulus locations. We sought to examine whether the effects observed in Experiment 2 would be present in these more complex search displays. A larger number of distractors leaves open the possibility that an AWB in these displays could involve attention to a single distractor or a group of distractors. While the results of Experiment 2 speak against an account of the AWB as a necessary component of a search and destroy process, Experiment 3 also provides an opportunity to further test this in larger set sizes, where the search and destroy approach involves an inherent penalty.

### Method

#### Participants

Sixteen participants took part in Experiment 3 (13 females; three males; *M*_age_ = 23.13 years; *SD* = 4.50; age range: 18–35 years). Recruitment, compensation, visual acuity, consent and ethical approval were subject to the same criteria as previous experiments.

#### Apparatus and stimuli

The apparatus and stimuli used in Experiment 3 were identical to those used in Experiment 2 with the following exceptions. One hundred and forty-four images of clocks and 144 images of keys were used as distractors (two blocks of 36 trials each contained displays with three distractors, one for each distractor category). In Experiments 3–5, the location of the pre-search stimulus was counterbalanced between all four possible display locations.

#### Procedure

The procedure was the same as for Experiment 2, with the following exceptions. There were two blocks where distractors were drawn from the category keys and two blocks where distractors were drawn from the category clocks. There was a blocked, within-participants manipulation of search display set size, such that in two blocks, displays contained three distractors and a target, and in two blocks, displays contained a single target and a single distractor. Distractor category and display set size were order counterbalanced.

### Results

#### Set size

To allow a similar analytic approach to that used before, we first adjusted the proportions of saccades to targets and distractors to allow comparisons between set sizes by multiplying proportion of first saccades to targets in set size four by three. One-sample *t*-tests (*μ* = 0.5 indicating no target/distractor bias, also see Fig. 2) revealed that there was a greater proportion of late (> 250 ms) first saccades made towards targets in both the two, *t*(15) = 3.89, *p* = .001, *d* = 0.97, and four set-size conditions, *t*(15) = 4.34, *p* < .001, *d* = 1.09. There was no target or distractor bias in early (< 250 ms) first saccades for either the two, *t*(15) = 0.06, *p* = .953, or four set-size conditions, *t*(15) = 1.37, *p* = .190.

#### Effects across set sizes

Repeating a similar ANOVA to that used in the previous experiments and including set size as a factor (within-participants: two, four) showed a main effect of first saccade latency, *F*(1,15) = 17.64, *p* < .001, η2_G_ = .25, with a smaller proportion of saccades made towards distractors in late compared to early saccades, but no main effect of set size, *F*(1,15) = 0.39, *p* = .542, and no interaction, *F*(1,15) = 0.09, *p* = .775.

#### Further exploratory analysis

We compared overall response times and first saccade latencies between set sizes in Experiment 3 (see Table 2). Response times, *t*(15) = 9.57, *p* < .001, *d* = 2.39, and first saccade latencies, *t*(15) = 2.19, *p* = .045, *d* = 0.55, were both significantly longer for displays with set size four compared to set size two. Continuing our previous approach, we also compared fixation durations between set sizes in Experiment 3 (see Table 2). Fixation durations were significantly longer for displays with set size two relative to four, and this effect was observed overall, *t*(31) = 8.05, *p* < .001, *d* = 1.42, for distractor fixations, *t*(31) = 3.04, *p* = .005, *d* = 0.54, and target fixations, *t*(31) = 3.33, *p* = .002, *d* = 0.59. To follow up on this, as might be expected, there was also a corresponding increase in the overall number of fixations made for displays of set size four (*M* = 4.10, *SD* = 1.48) relative to set size two (*M* = 2.70, *SD* = 0.82; *t*(31) = 10.29, *p* < .001, *d* = 1.82).

### Discussion

These findings paralleled those of Experiment 2 and showed that effective proactive suppression of attention to distractors was similar in displays with one and three distractors. For displays with three distractors relative to one, response times and first saccade latencies were longer, but fixations more frequent and shorter in duration, suggesting that the slowing of response times was driven by slower initial guidance and the need to process more stimuli for the larger set size. Compared to Experiment 2, the timing of the proactive suppression observed here under ignore instructions suggests that the location of the pre-search stimulus relative to the target was not a necessary condition of the elimination of the AWB effect observed in Experiment 1, but that it likely influenced the speed of distractor suppression via inhibition of return to the distractor location on a proportion of trials.

With only a single distractor category present in each block of Experiment 3, we cannot rule out that learning about distractors influenced participants’ performance within blocks. However, such effects have previously been observed over much longer blocks in tasks with simpler colour stimuli and fixed distractor colours (Cunningham & Egeth, 2016). We expect that in the present case, any such effect would be minimised by the short block length, complex stimuli, and variation in distractor category between blocks. The pattern of first saccades in larger set size with three distractors shows that first saccades to targets become more frequent than first saccades to distractors at approximately 200 ms, providing evidence of limited parallel distractor suppression, rather than serial search and rejection. To further explore the influence of category specificity on the effect of the pre-search stimulus, we examined the effect of category congruency between the pre-search stimulus and distractors in Experiment 4.

## Experiment 4

Experiment 4 included a manipulation of congruency between the pre-search stimulus and distractor, such that the pre-search stimulus could be drawn from the same category as the distractor or a task-irrelevant category. We previously suggested that the distractor-congruent pre-search stimuli in Experiments 2 and 3 eliminated the AWB effect because they disrupted early attentional guidance from distractor templates held in WM. We predicted that this would rely on category congruency between the pre-search stimulus and distractor and expected to once again observe an AWB, as in the ignore condition of Experiment 1, when the pre-search stimuli and distractor categories were incongruent.

### Method

#### Participants

Sixteen participants took part in Experiment 4 (13 females; three males; *M*_age_ = 24.69 years; *SD* = 4.05; age range: 20–33 years). Recruitment, compensation, visual acuity, consent and ethical approval were subject to the same criteria as previous experiments.

#### Apparatus and stimuli

The apparatus and stimuli used in Experiment 4 were identical to those used in Experiment 2. Pre-search stimuli were either congruent or incongruent with the distractor category (randomly interleaved trials, 50% within each block). Incongruent pre-search stimuli were selected from a range of other object categories and never matched either the target or distractor category.

#### Procedure

The procedure was the same as for the ignore instructions condition in previous experiments with the exception of the within-participants pre-search stimulus congruency manipulation (see *Apparatus and stimuli* above).

### Results

#### Pre-search stimulus congruency

**Fig. 4:**
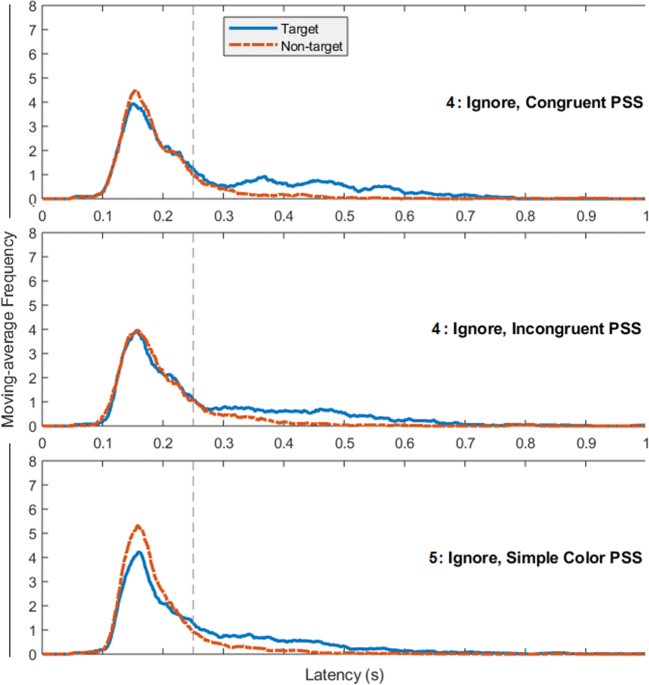
Moving-average frequency of first saccades made to targets and distractors in Experiments 4 and 5, calculated over onset latencies for all first saccades. The top and middle rows show data from Experiment 4, which included pre-search stimuli (PSS), which were either congruent or incongruent with the distractor category. The bottom row shows data from Experiment 5, where a simple colour PSS was used

We used the same analytic approach as in previous experiments (see Figs. 3 and 4). One-sample *t*-tests (*μ* = 0.5 indicating no target/distractor bias) revealed that a greater proportion of late (> 250 ms) first saccades were made towards targets in both congruent, *t*(15) = 3.84, *p* = .002, *d* = 0.96, and incongruent trials, *t*(15) = 5.21, *p* < .001, *d* = 1.30. In contrast, early (< 250 ms) first saccades showed no target or distractor bias in congruent, *t*(15) = 0.39, *p* = .703, or incongruent trials, *t*(15) = 0.37, *p* = .714.

#### Effects across pre-search stimuli

Including first saccade latency and pre-search stimulus congruency (congruent or incongruent) as within-participants factors in an ANOVA revealed a main effect of first saccade latency, *F*(1,15) = 38.17, *p* < .001, η2_G_ = .341, no effect of congruency, *F*(1,15) = 0.01, *p* = .908, and no interaction, *F*(1,15) = 0.01, *p* = .906.

#### Further exploratory analysis

We also compared overall response times and first saccade latencies between congruent and incongruent pre-search stimuli in Experiment 4 (see Table 2). There was no effect of pre-search stimulus congruency on response time, *t*(15) = 1.15, *p* = .269, or first saccade latency, *t*(15) = 0.14, *p* = .888. We again compared fixation durations between congruent and incongruent pre-search stimuli in Experiment 4 (see Table 2). Target fixations were significantly longer when the PSS was congruent, *t*(15) = 2.23, *p* = .041, *d* = 0.56, but distractor fixations were significantly longer when the PSS was incongruent, *t*(15) = 2.48, *p* = .026, *d* = 0.62. There was no effect on overall fixation durations, *t*(15) = 1.13, *p* = .275. Follow-up analysis revealed no differences between the congruent and incongruent PSS conditions in the number of fixations made on targets (*M*_*cong*_ = 1.62, *SD*_*cong*_ = 0.36; *M*_*incong*_ = 1.65, *SD*_*incong*_ = 0.33; *t*(15) = 1.01, *p* = .327) or distractors (*M*_*cong*_ = 1.23, *SD*_*cong*_ = 0.18; *M*_*incong*_ = 1.26, *SD*_*incong*_ = 0.16; *t*(15) = 1.57, *p* = .137).

### Discussion

Contrary to our predictions, our analysis suggested no effect of pre-search stimulus category congruence on the AWB – the observed effect was the same irrespective of the category of the pre-search stimulus. These results indicated that the elimination of the AWB effect we previously observed did not depend upon category congruence or a specific predictive relationship between the pre-search stimulus and distractor.

While both congruent and incongruent pre-search stimuli were sufficient to reduce guidance towards distractors and eliminate the AWB effect we observed in Experiment 1, our exploratory analysis of fixation durations suggested a small congruency effect on later distractor and target recognition. Congruent pre-search stimuli modestly increased target fixation durations, suggesting slower target recognition, whereas incongruent pre-search stimuli modestly increased distractor fixation durations, suggesting slower distractor recognition. In short, the category-specific informational content of a photographic pre-search stimulus had no impact on initial attention (as measured by first saccades here), but it did appear to influence the recognition of both targets and distractors.

One explanation of these results is that pre-search stimuli broadly disrupt guidance from distractor templates, which would otherwise drive initial attention toward the distractor, and that any photographic pre-search stimulus, as a collection of complex visual features, is sufficient to achieve this effect. While the full bottom-up feature-based analysis of our pre-search stimuli necessary to fully disambiguate these results is beyond the scope of the present study, we tested this account in Experiment 5 by examining whether simple uniformly coloured pre-search stimuli would also disrupt the AWB.

## Experiment 5

Experiment 4 demonstrated that the specific category relationship between the pre-search stimuli and distractors did not influence the disruption of the AWB effect. Experiment 5 explored the extent to which this generalised further, specifically, whether a simple colour pre-search stimulus could have the same effect. We considered two possible outcomes here: Firstly, if pre-search stimulus simply causes a feature-agnostic delay in allocating attention to the search display, then the same results should be observed as in Experiment 4. Alternatively, if the complex visual features of a photographic pre-search stimulus, regardless of the specific category, are necessary to eliminate the AWB effect we had previously observed, we would expect a simple colour pre-search stimulus not to do so.

### Method

#### Participants

Sixteen participants took part in Experiment 5 (12 females; four males; *M*_age_ = 23.13 years; *SD* = 4.83; age range: 19–36 years). Recruitment, compensation, visual acuity, consent and ethical approval were subject to the same criteria as previous experiments.

#### Apparatus and stimuli

The apparatus and stimuli used in Experiment 5 were identical to those used in Experiment 2, with the exception that pre-search stimuli were uniformly red- or blue-coloured squares (CIE xyY [.343, .335, 99.37] and [.289, .313, 106] respectively).

#### Procedure

The procedure was the same as for the ignore instructions condition in Experiment 2 with the following exceptions. There was only a single distractor category per block and for each participant the colour of the pre-search stimulus (red or blue) reliably predicted a distractor category (keys or clocks; counterbalanced).

### Results

#### Simple colour pre-search stimulus

We used the same analytic approach as in previous experiments. One-sample *t*-tests (*μ* = 0.5 indicating no target/distractor bias, also see Fig. 2) showed that a greater proportion of early (< 250 ms) first saccades were made towards the distractor, *t*(15) = 2.76, *p* = .015, *d* = .690), and a greater proportion of late (> 250 ms) first saccades were made towards the target, *t*(12) = 3.55, p = .004, d = 0.98 (three participants did not make any late first saccades and were not included in this analysis).

#### Complex and simple pre-search stimuli

To examine the effect of a simple colour pre-search stimulus relative to a complex photographic, we conducted an additional comparison between early (< 250 ms) first saccades in Experiments 2 and 5 (see Table 2). The proportion of early first saccades towards the distractor was significantly lower in Experiment 2 (with a complex photographic pre-search stimulus), than in Experiment 5 (simple colour pre-search stimulus), *t*(30) = 4.82, *p* < .001, *d* = 1.70.

#### Further exploratory analysis

We compared overall response times and first saccade latencies between Experiments 2 and 5 (see Table 2, *p*-values Bonferroni-corrected for the three exploratory comparisons on these data) and found no significant differences in response time, *t*(30) = 1.25, *p* = .663, or first saccade latency, *t*(30) = 0.75, *p* > .999. We again compared fixation durations between Experiments 2 and 5 (see Table 2), and found no differences in overall, *t*(30) = 0.31, *p* > .999, distractor, *t*(30) = 0.13, *p* > .999, or target, *t*(30) = 0.82, *p* > .999, fixation durations.

There was no difference in the overall response time between the ignore condition of Experiment 1 and Experiment 5, *t*(30) = 1.27, *p* = .642, but the first saccade latency was significantly slower in Experiment 5, *t*(30) = 3.10, *p* = .012, *d* = 1.10. There were no differences in fixation duration between the ignore condition of Experiments 1 and 5 for overall, *t*(30) = 1.19, *p* = .243, distractor, *t*(30) = 0.27, *p* > .999, or target, *t*(30) = 1.83, *p* = .234, fixation durations.

### Discussion

The results of Experiment 5 demonstrate that the presentation of a simple colour pre-search stimulus was not sufficient to eliminate the AWB effect we had previously observed. Here we found a pattern of first saccades that matched the ignore condition of Experiment 1 (no pre-search stimulus), but similar response times to Experiments 2 and 4 (with a pre-search stimulus). To link these results to the possible explanations we offered above, the presence of an AWB effect and a slower average first saccade latency than observed in Experiment 1 strongly suggest that just slowing initial attention is not sufficient to eliminate the AWB. Instead, it appears that the simple colour pre-search stimulus lacked the complex features of the photographic stimuli used in the preceding experiments, and it could be processed and/or ignored rapidly without disrupting initial guidance to the search display.

## General discussion

Experiment 1 provided evidence for the AWB effect, unintended shifts of attention towards distractors, during visual search of photographic real-world objects. Importantly, the search task used here allowed us to provide participants with broad distractor cues, while selecting targets from an unspecified and unpredictable set of categories. This ensured that when participants were cued with a distractor category, this was the only source of guidance and that inferring or predicting target categories was not possible. We observed near-identical patterns of initial saccades between participants who were explicitly instructed to ignore distractors and those who were explicitly instructed to find those same stimuli. Accordingly, under these standard conditions in Experiment 1, a reactive search and destroy mechanism provides a precise and parsimonious account of participants’ early attentional biases as reflected in the first saccades made when trying to ignore distractors.

In Experiment 2, a pre-search stimulus strikingly eliminated the AWB effect we had previously observed (a result that was replicated in Experiments 3 and 4). Where an AWB was present in Experiment 1 (early first saccades), it was absent, and instead first saccades were more likely to be made towards the target. If an AWB were a necessary initial stage of distractor suppression, as in a search and destroy process, then not reactively finding and eliminating distractors should have disrupted both distractor suppression and attention to targets. Instead, we observed improved distractor suppression, consistent with an account of the AWB as a failure of proactive suppression linked to distractor representations held in WM.

### Effects of the pre-search stimulus

The AWB we observed in Experiment 1 was consistent with evidence that the contents of WM can bias attention towards matching objects irrespective of task (Han & Kim, 2009; Sawaki & Luck, 2011; Woodman & Luck, 2007). This AWB effect was then eliminated following the presentation of a pre-search stimulus in Experiments 2–4, which reduced early first saccades towards the distractor, consistent with the disruption of guidance toward the distractor category that participants were holding in WM. However, an AWB returned in Experiment 5 when the pre-search stimulus was a simple colour, coupled with a slower first saccade latency than in Experiment 1, suggesting that the pre-search stimulus did not simply eliminate the AWB via a *general* disruption or slowing of early saccades.

These findings suggest that the pre-search stimulus functioned to selectively disrupt the categorical distractor representations that guided attention towards distractors. This has some similarity to within-trial distractor preview effects, where improved suppression was observed when basic distractor features matched previewed features (for a review, see Olivers et al., 2006). While the specific category of the pre-search stimulus appeared to influence later recognition, it did not influence initial guidance, and the pre-search stimulus did not eliminate the AWB effect when it was a simple colour (response times remained broadly consistent where there was no set size manipulation or find instruction). The complex visual features of a briefly presented photographic pre-search stimulus, relative to a simple colour, will inevitably have higher similarity to the visual features associated with a categorical distractor cue, irrespective of distractor category congruency. It is therefore likely that following a broad categorical distractor word cue that does not precisely specify particular visual features, any complex photographic pre-search stimulus is processed in a way that disrupts early attention to distractors and promotes proactive suppression. It is therefore possible that the pre-search stimulus might not have the same effect if participants were cued to ignore specific distractors rather than broad distractor categories.

A consistent pattern associated with the presence of a pre-search stimulus in Experiments 2, 4 and 5, including the simple colour stimulus, was that approximately 30% of first saccades were late (> 250 ms onset latency), up from under 10% in Experiment 1 where there was no pre-search stimulus (see Table S1 in OSM). Experiment 3 had a more even split between early and late first saccades, likely due to the order counterbalanced and blocked set size manipulation resulting in slower first saccades overall. If the explanation that we offer is correct, then the disruption caused by the pre-search stimulus must take some additional time to resolve and cause slower first saccades. However, the results of Experiment 5, where an AWB was observed alongside first saccade latencies and response times similar to Experiment 2, show that presence of a pre-search stimulus slows down first saccades, but that this is not sufficient to eliminate the AWB effect.

### Proactive suppression: Habitual versus flexible

It has previously been reported that with prolonged practice the AWB can be eliminated and reversed in behavioural measures (Cunningham & Egeth, 2016). This finding is related to research on habitual processes that shows over many trials distractors can be effectively suppressed without effortful attention to them (e.g., Moorselaar & Slagter, 2019) – in everyday searches such processes may be more important than flexible, voluntary suppression. Physiological evidence also points to effective suppression of the AWB over time both when distractors vary trial-by-trial and when they are held constant over blocks of trials (Gaspelin & Luck, 2018a; Sawaki et al., 2012). While habitual resistance to the AWB is more robust with consistent distractors, tasks with variable distractors may still tap into highly-practised cue-distractor processing (Theeuwes, 2013), rather than flexible, unpractised responses evident in the first few trials of an experiment.

Our analysis of target and distractor biases in first saccades across experiments provides evidence that, in addition to powerful habitual and stimulus-driven mechanisms that overcome the AWB, attention can be flexibly guided away from distractors and towards targets without extensive practice. While previous studies report elimination of the AWB of many hundreds of trials, we demonstrate a similar effect over comparatively few trials by disrupting initial distractor biases with the presentation of a pre-search stimulus.

In conclusion, the current findings provide evidence that initial attention toward distractors is *not* a necessary stage in effective voluntary distractor suppression, and that it can be attenuated without adverse effects on attentional guidance. This is inconsistent with search and destroy accounts of the AWB, which otherwise provides a parsimonious and intuitive account of a puzzling phenomenon. The AWB is likely a by-product of distractor templates held in WM that guide attention to matching items even when these are irrelevant. Our findings show that this process can be disrupted, weighing decisively in favour of the AWB as a failure of proactive suppression. While AWB effects are not observed in *all* visual search tasks, these findings parallel similar effects in perception, memory and motor processes, which likely also reflect failed attempts at suppression. Understanding failures in suppression more fully in visual attention may aid future developments around similar effects in other domains.

## Supplementary Information

ESM 1 (DOCX 410 kb)

## Data Availability

The data and materials that support the findings of this study are available from the corresponding author upon reasonable request.
