# Supplementary material for: Attenuating the ‘attentional white bear’ effect enhances suppressive attention
Source: Atten Percept Psychophys. 2022 Sep 22;84(8):2444–60. doi: 10.3758/s13414-022-02560-w (PMC9630199; doi:10.3758/s13414-022-02560-w)
Supplement: Supplementary file 1 — (DOCX 410 kb) [file 13414_2022_2560_MOESM1_ESM.docx]

Eliminating the ‘Attentional White Bear’ Effect Enhances Suppressive Attention

Alex Muhl-Richardson, Maria Tortosa-Molina, Sergio A. Recio

Maximilian G. Parker and Greg J. Davis

**Supplementary Material**

**Full ‘Ignore’ Instructions**

1. A fixation cross will appear in the centre of the screen.

2. Click the fixation cross. **One picture will briefly appear. Ignore this completely.**

3. Then two pictures will appear simultaneously around the fixation cross. These pictures can appear to the right of, left of, above or below the fixation cross.

4. One picture will be a CLOCK, please TRY TO IGNORE THIS and click the other picture.

5. There will be different pictures in every trial. One will always be a CLOCK (or watch), ignore this and click on the other picture.

Please work as quickly and accurately as possible.

*Note: Text in* *bold in instruction number two was only shown for experiments with a pre-search stimulus; in instructions four and five the category “CLOCK” was replaced with other categories and other minor variations were made as described in the experimental methods.*

**Full ‘Search and Destroy’ Instructions**

1. A fixation cross will appear in the centre of the screen.

2. Click the fixation cross. **One picture will briefly appear. Ignore this completely.**

3. Two pictures will appear simultaneously around the fixation cross. These pictures can appear to the right of, left of, above or below the fixation cross.

4. One picture will be a CLOCK, please FIND THIS and then click the other picture.

5. There will be different pictures in every trial. One will always be a CLOCK (or watch); find this and click on the other picture.

Please work as quickly and accurately as possible.

*Note: Text in* *bold in instruction number two was only shown for experiments with a pre-search stimulus; in instructions four and five the category “CLOCK” was replaced with other categories and other minor variations were made as described in the experimental methods.*

**Additional Analysis and Data Pooling for Experiments 1 and 2**

In Experiments 1 and 2 the number of distractor categories per block was manipulated within-subjects. Of the four blocks, two were *one-category* blocks, involving only clocks or only keys as distractors. The other two blocks were *two-category* blocks, each comprising unpredictably interleaved trials with clock or key distractors. While the one-category blocks were of primary importance, giving rise to the clearest predictions from theory and previous work, we also examined differences in first saccades between one- and two-category blocks. In Experiment 1, a mixed two-way ANOVA on the proportion of first saccades to distractors, with number of distractor categories (within-participants: one, two) and instruction (between-participants: ignore, search and destroy) found no main effect of number of distractor categories, *F* = 0.13, and no interaction with instruction, *F* = 0.11. Identical analyses for Experiment 2, similarly yielded no effects of number of distractor categories, *F* = 1.54, and no interaction with instruction, *F* = 2.51. Accordingly, our analyses in the main text collapsed across this factor, maximising our power particularly with regard to late first saccades and providing a simple description of those results consistent with subsequent experiments that involved only one-category blocks.

**Proportion of Early and Late First Saccades**

Table S1

*Mean Proportions of Early (<250 ms onset latency) and Late (>250 ms onset latency) First Saccades for All Experiments and Conditions*

| Experiment and Condition | Mean Proportion of Early First Saccades | Mean Proportion of Late First Saccades |
| --- | --- | --- |
| E1 Ignore | 0.92 (0.12) | 0.08 (0.12) |
| E1 Find | 0.94 (0.11) | 0.06 (0.11) |
| E2 Ignore (PSS) | 0.65 (0.32) | 0.35 (0.32) |
| E3 Set size 2 | 0.48 (0.35) | 0.52 (0.35) |
| E3 Set size 4 | 0.43 (0.31) | 0.57 (0.31) |
| E4 Congruent PSS | 0.70 (0.32) | 0.30 (0.32) |
| E4 Incongruent PSS | 0.69 (0.32) | 0.31 (0.32) |
| E5 Colour PSS | 0.71 (0.29) | 0.29 (0.29) |
| Note: parentheses show standard deviations. | | |

**Experiment S1 – Baseline**

The clear distractor bias in early (<250 ms) first saccades in Experiment 1 was interpreted as a paradoxical consequence of participants’ *top-down* attempts to ignore those distractors. It could be argued that this bias might reflect greater *bottom-up* salience of distractors relative to targets, or some other stimulus factor. To exclude this possibility, we conducted a baseline experiment (S1) that presented *exactly* *the same stimuli* in each display as Experiment 1, but employed a *different task* that did not require observers to ignore either target or distractor stimuli.

**Method**

**Observers.** Sixteen observers took part in Experiment S1 (6 females; 10 males; *M*_age_ = 24.81 years; *SD* = 5.91; age range: 18-38 years). Recruitment, compensation, visual acuity, consent and ethical approval were subject to the same criteria as in previous experiments. One observer’s data were excluded from analysis due to an error synchronising eye movement and behavioural data.

**Apparatus and stimuli.** The apparatus and stimuli used were identical to those used in Experiment 1 with the following exceptions. An extra set of stimuli was created, in which for each display, four identical square Gabor patches on grey square backgrounds (25% opacity and 0.55° of visual angle) were inserted into the four corners of both pictures displayed, such that one of the two stimuli contained four vertical patches and the other, four horizontal patches.

**Procedure.** The procedure was the same as for Experiment 1, with the following exceptions. Observers were instructed that on each trial, 500 ms after the onset of the photographic images, the small Gabor patches would appear on each stimulus for 100 ms before offsetting (all timing was otherwise identical to Experiment 1). The patches on one image would be vertical, and on the other, horizontal; the task was simply to select the image with horizontal patches by clicking on it. The size and high spatial frequency of the Gabor patches was intended to make this impossible if observers kept their gaze at fixation. However, the task would be fairly simple if the observer moved their gaze toward one of the two images prior to the onset of the patches. Observers were instructed to do this, and informed that, though the contents of the photographic stimuli provided no information as to which stimulus would comprise the horizontal patches, they could perform the task using the following logic. If they happened to move their gaze to the image on which horizontal patches subsequently appeared, they could click on that image, whereas if they happened to move their gaze to the image on which vertical patches appeared, they could simply click on the *other* image, knowing that must have had horizontal patches. Accordingly, provided that they moved their eyes quickly to one image or the other, they could perform the task. This simple task was intended to encourage observers to make saccades toward the images, but not to involve any instruction to ignore any image category.


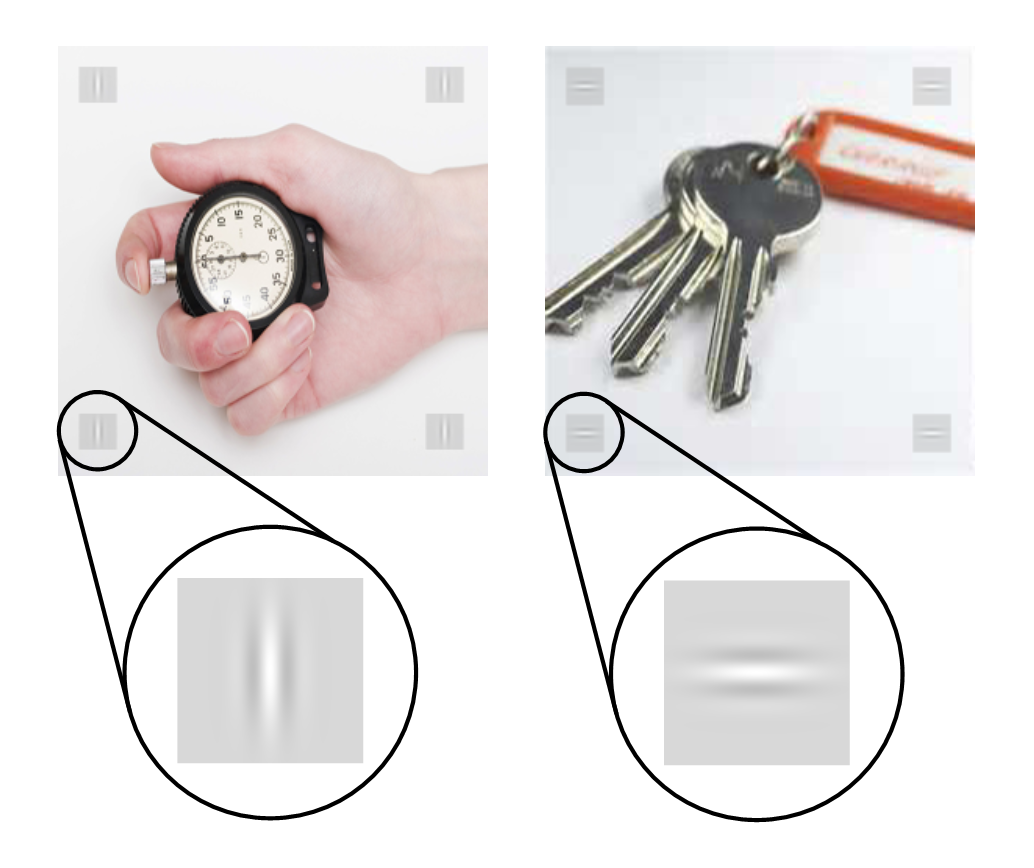


*Figure S1. Typical images employed in the Baseline Experiment S1. Note that all such images were identical to those in Experiment 1 for the first 500 ms of the display, following this small Gabor patches were briefly added (for 100 ms).*

**Results**

Figure S2 plots the distractor bias in early (<250 ms) first saccades from the ignore instruction condition in Experiment 1 next to that for identical stimuli in Experiment S1. Visual inspection of the plot suggested that there was little or no distractor bias in the Baseline Experiment S1. We conducted a two-way, mixed ANOVA on the proportion of early first saccades made to distractors, with experiment (between-subjects: Experiment 1 ignore instructions, Baseline Experiment S1) and distractor categories per block (within-subjects: one, two) as factors. This yielded a main effect of experiment, *F*(1,29) = 8.64, *p* < .006, η^2^_G_ = .139, reflecting a greater bias towards distractors under ignore instructions in Experiment 1 than the Baseline Experiment S1. There was no main effect of the number of distractor categories per block, *F* = 0.22, and no interaction, *F* = 2.42.

These findings provide strong confirmation that the AWB observed in Experiment 1 under ignore instructions indeed reflected participants’ attempts to ignore stimuli in those conditions, not any intrinsic differences in the stimuli *per se*. They also did not provide clear evidence of differences as a function of one- versus two- distractor categories. However, we were reassured that any numerical trend was particularly compelling for one-distractor conditions, of primary importance as they given that they were common to all experiments here and gave rise to the clearest predictions from theory.


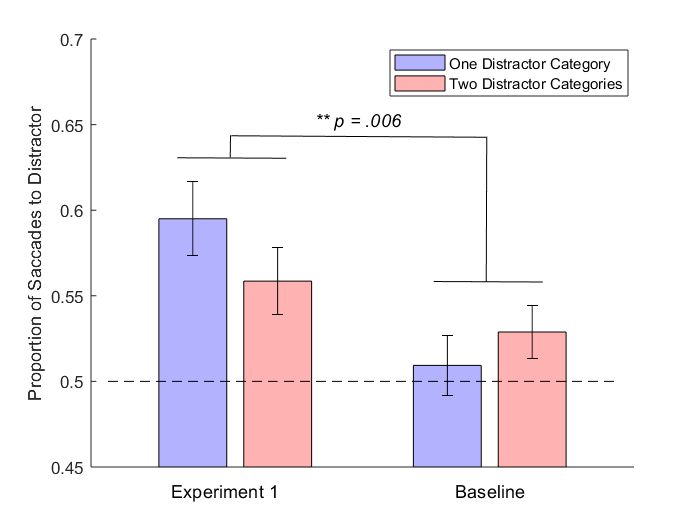


*Figure S2. Proportion of initial, shorter-latency saccades toward distractors in Experiments 1 (ignore instructions) and S1 (baseline), separately for trials with one versus two distractor categories.*
